# A Combined Data-Driven and Model-Based Algorithm for Accurate Battery Thermal Runaway Warning

**DOI:** 10.3390/s24154964

**Published:** 2024-07-31

**Authors:** Qingyang Chen, Yinghui He, Nengjie Fang, Guanding Yu

**Affiliations:** 1 College of Information Science and Electronic Engineering, Zhejiang University, Hangzhou 310027, China; 22231156@zju.edu.cn (Q.C.); 2014hyh@zju.edu.cn (Y.H.); 2 Hangzhou Zhonhen Electric Co., Ltd., Hangzhou 310053, China; fangnj@hzzh.com

**Keywords:** thermal runaway warning, model-driven, data-driven, K-Means, Bernardi equation

## Abstract

With the increasingly widespread application of large-scale energy storage battery systems, the demand for battery safety is rising. Research on how to detect battery anomalies early and reduce the occurrence of thermal runaway (TR) accidents has become particularly important. Existing research on battery TR warning algorithms can be mainly divided into two categories: model-driven and data-driven methods. However, the common model-driven methods are often of high complexity, with poor versatility and low early warning capability; and the common data-driven methods are mostly based on neural networks, requiring substantial training costs, with better early warning capabilities but higher false alarm probabilities. To address the limitations of existing works, this paper proposes a combined data-driven and model-based algorithm for accurate battery TR warnings. Specifically, the K-Means algorithm serves as the data-driven module, capturing outliers in battery data, and the Bernardi equation serves as the model-driven module used to evaluate battery temperature. Ultimately, the outputs of the weighted model-driven module and data-driven module are combined to comprehensively assess whether the battery is abnormal. The proposed algorithm combines the advantages of model-driven and data-driven approaches, achieving a 25 min advance warning for thermal runaway, with a significantly reduced probability of false alarms.

## 1. Introduction

### 1.1. Background

With the popularization of electric vehicles, renewable energy storage systems, and mobile devices, the application of large-scale energy storage battery systems is becoming increasingly widespread [1]. Their application covers various fields, including transportation, energy storage, and mobile communications. At the same time, the application of renewable energy storage systems promotes the utilization and storage of clean energy, reducing reliance on traditional energy sources and achieving sustainable development [2]. However, the widespread use of large-scale energy storage battery systems has also brought about many challenges and demands. One of these is the increasing demand for battery safety [3]. Due to the potential generation of heat during battery charging, discharging, or prolonged use, overheating, explosions, fires, and other hazardous situations may occur. Therefore, research on how to detect battery anomalies early and issue battery thermal runaway (TR) warnings as soon as possible to reduce the occurrence of TR accidents has become particularly important.

Existing research on battery TR warning algorithms can be mainly divided into two categories: model-driven and data-driven methods. Model-driven methods involve establishing physical models of the batteries themselves, such as electrochemical models, thermodynamic models, etc., to predict internal changes in the battery and achieve TR warnings [4]. Data-driven methods, on the other hand, utilize neural networks, machine learning, and other methods to analyze massive characteristic parameters generated by batteries to determine if anomalies have occurred [5]. Model-driven methods are often complex with poor versatility, capable of issuing warnings only for a specific type of TR in an individual battery cell, and they also provide relatively short lead times for warnings, leaving insufficient time for preventive responses. In contrast, common data-driven methods typically involve neural networks, requiring extensive pre-training and parameter adjustments, but they significantly improve the lead time for warnings compared to model-driven methods. However, they are more susceptible to false alarms.

In summary, both model-driven and data-driven methods have their advantages but also exhibit certain drawbacks. Therefore, there is an urgent need to propose a battery TR warning method driven by both model and data to achieve complementary advantages.

### 1.2. Related Works

There have been several studies on battery thermal runaway algorithms based on either model-driven or data-driven approaches.

From a model-driven perspective, Ouyang et al. [6] established an electrochemical–thermal coupling model to predict the voltage and temperature of lithium batteries during thermal runaway. This model accurately describes the electrochemical thermodynamic couplings in the context of battery capacity degradation under high temperatures induced by internal short circuits (ISC), reflecting the degradation of the solid electrolyte interface at extreme temperatures. Dey et al. [7] proposed a lithium battery thermal fault diagnosis scheme based on a partial differential equation (PDE) electrochemical model. They utilized a distributed parameter one-dimensional thermal model for cylindrical batteries and PDE observer-based techniques to achieve real-time detection and estimation of ISC within the battery. Wei et al. [8] introduced a battery fault detection method using multiple EECM models and a strong tracking Kalman filter. This approach estimated the future trend of the battery’s internal resistance using the Kalman filter, identifying moments of significant increase or decrease in resistance as instances of battery faults.

In terms of data-driven methods, due to their high prediction accuracy and low requirements for system models, more and more research teams are attempting to use data-driven methods to achieve battery TR warning. Zhang et al. [9] utilized an electro-thermal coupled simulation model to generate data for training neural networks. They established a conventional Long Short-Term Memory (LSTM) neural network and a Multi-Mode Multi-Task Predictive Neural Network (MMTPFNN), capable of issuing a TR warning 200 s in advance. Li et al. [10] combined Long Short-Term Memory (LSTM) and Convolutional Neural Network (CNN) to propose a TR prediction model based on Abnormal Heat Generation (AHG). This approach accurately predicted battery temperatures for 48 time steps and achieved a 27 min lead time for TR predictions. Zhang et al. [11] proposed a rapid and efficient multi-fault diagnosis method for lithium–ion batteries, leveraging curvilinear Manhattan distance and voltage difference analysis. Experimental outcomes underscore its capability to detect and isolate multiple faults with remarkable sensitivity and reliability, while achieving a low computational expense and unparalleled accuracy. Li et al. [12] employed the K-Shape clustering algorithm to cluster the temperature time-series data of individual battery cells within a battery pack. By evaluating the similarity before and after clustering, they determined the presence of TR indications, enabling a 90 min advance warning compared to traditional Battery Management Systems (BMS). In addition, there are many other methods, such as DBSCAN [13], MKELM [14], LSSVM [15], and so on.

### 1.3. Motivations and Contributions

Overall, the majority of current battery TR warning algorithms are confined to either model-driven or data-driven approaches. Although these algorithms have achieved partial performance improvements, they have not addressed the fundamental issues inherent in single-driven approaches. Present research on model-driven approaches is generally limited to fault detection, without considering the time interval between fault detection and the occurrence of TR, thus failing to prove the adequacy of the algorithms’ lead time for early warning. Furthermore, most model-driven algorithms are only applicable to a specific type of TR fault, such as internal short circuit or external short circuit, leading to low versatility. In terms of data-driven approaches, they often incur significant training costs, requiring a large amount of training data and extensive time for parameter tuning. Although data-driven approaches have improved the lead time for TR prediction, there is minimal research delving into the false alarm probability of such algorithms.

To overcome the limitations of existing works, in this paper, we propose a combined data-driven and model-based algorithm for accurate battery TR warning. By integrating the K-Means algorithm with the Bernardi equation, a synergistic complementarity between data-driven and model-driven approaches is achieved, ensuring the high versatility and low training costs of the algorithm. Additionally, we enhance the performance of the algorithm, conducting detailed experiments on its lead time and accuracy in TR prediction. The results indicate that the proposed algorithm can provide a 25-min advance warning for TR with a low probability of occurrences of false alarms or missed warnings. The main contributions of this paper are summarized as follows.

- A combined data-driven and model-based algorithm is proposed to realize accurate battery TR warning. The data-driven component employs the K-Means clustering algorithm to cluster the temporal state data of batteries. The anomaly is detected by observing abrupt changes in the clustering results. On the other hand, the model-driven component, facilitated by the Bernardi equation, utilizes the battery’s charge and discharge currents to predict the battery’s temperature under normal conditions. By comparing this prediction with the actual temperature, it assesses whether the battery temperature is abnormal.
- Taking into account the strengths and weaknesses of model-driven and data-driven approaches in the context of TR prediction, this study employs a parallel fusion approach to integrate both methods. Initially, the K-Means algorithm is used to assess the battery’s state, and if an anomaly is detected, the Bernardi algorithm is introduced for a secondary assessment. By employing support vector machines to integrate the output parameters of both methods, the alarm threshold is determined.
- Two sets of single-cell battery TR data and two sets of battery pack TR data are used to test the algorithm’s performance. The lead time and accuracy of TR prediction for the model-driven approach, data-driven approach, and the proposed algorithm are tested separately. The experimental results indicate that the proposed algorithm exhibits high versatility and low training costs. They also verify the superior lead time compared to the model-driven approach and the low probability of false alarms compared to the data-driven approach, showcasing outstanding performance.

### 1.4. Organization

The rest of this paper is organized as follows. In Section 2, the fundamental principles of the K-Means clustering algorithm and the Bernardi equation, as well as the mechanism for TR prediction, are comprehensively expounded. Section 3 provides a detailed exposition of the double-driven TR prediction algorithm proposed in this study, including the double-driven fusion method, result weighting. Section 4 meticulously outlines the experimental procedure for TR and the analysis results of the algorithm. Finally, Section 5 concludes the whole paper.

## 2. Theoretical Principles

In this section, we provide a detailed introduction to the principles of the Bernardi equation and the K-Means clustering algorithm.

### 2.1. Bernardi Equation

Temperature is a critical indicator for monitoring the TR behavior of batteries. Predicting the temperature change trend of batteries under normal operating conditions can offer valuable data for detecting TR during actual use. To accomplish this objective, it is essential to establish the relationship between electrical parameters such as battery charge/discharge current, charge/discharge voltage, open circuit voltage, and battery temperature. To this end, we utilize the Bernardi equation to fulfill this task and develop a conversion model between battery electrical energy and thermal energy [16]. The Bernardi equation can be derived and comprehended in the following simplified manner.

Assuming that the temperature of the battery changes ΔT during charging or discharging, the required heat *Q* for this process can be expressed as

(1) Q=CmΔT,

where *C* represents the specific heat capacity of the battery and *m* denotes the mass of the battery. The heat of batteries is related to heat dissipation and production. The disparity between heat dissipation $Q_{dis}$ and heat generation $Q_{gen}$ determines the net heat *Q* gained or lost by the battery. This relationship can be expressed using the following formula

(2) $$Q=Q_{dis}+Q_{gen}.$$

Specifically, due to the fact that the operational temperature of batteries is usually higher than the ambient temperature, heat dissipation primarily occurs through the exchange of heat between the battery and its surroundings. Specifically, the calculation formula for $Q_{dis}$ can be expressed as:

(3) $$Q_{dis}=h\left(T_{amb}-T\right)\Delta t.$$

In particular, *h* represents the heat dissipation coefficient between the battery and the surrounding environment, $T_{amb}$ denotes the ambient temperature, *T* is the battery temperature, and δt denotes time.

The heat release of a battery is caused by internal chemical reactions during the charging and discharging process of the battery, which can be calculated using the following formula

(4) $$Q_{gen}=\left[I\left(V-U\right)+IT\frac{dU}{dT}\right]\Delta t,$$

where *I* represents the charging or discharging current of the battery, *V* is the charging or discharging voltage of the battery, *U* denotes the open circuit voltage of the battery, and $\frac{dU}{dT}$ is the entropy heat coefficient of the battery.

By combining the above equations, the thermodynamic model of the battery, namely the Bernardi equation, can be obtained as

(5) $$Cm\frac{dT}{dt}=h\left(T_{amb}-T\right)+I\left(V-U\right)+IT\frac{dU}{dT}.$$

The Bernardi equation can establish the relationship between the battery’s electrical parameters and thermal parameters. Meanwhile, in contrast to complex electrochemical–thermodynamic coupling models, the characteristics of the battery model required by the Bernardi equation can be obtained through relatively simple experimental measurements. Specifically, we need to identify the specific heat capacity, mass, heat dissipation coefficient, voltage, and current of the battery. However, the open circuit voltage and entropy heat coefficient of the battery cannot be dynamically measured during battery operation. Nevertheless, these two parameter have a one-to-one correspondence with the battery state of charge (SOC), and thus can be derived from the SOC during battery operation. Thus, the actual temperature can be estimated using the Bernardi equation. If the actual temperature of the battery significantly exceeds the predicted value of the Bernardi equation, the battery is highly likely to undergo thermal runaway.

### 2.2. K-Means Clustering Algorithm

The K-Means algorithm originated from a vector quantization method in signal processing and has gained popularity as a clustering analysis method in the field of data mining. Given an observation set $\left\{x_{1},x_{2},x_{3},\cdots ,x_{n}\right\}$ where each observation is a *d*-dimensional real vector, the K-Means clustering aims to partition these *n* observations into *k* clusters (k≤n), and the summation of distances between each point and its corresponding cluster center is minimized.

The process of the K-Means clustering algorithm can be given as follows [17]:

(a) Initialization: randomly select *k* observations from the dataset as initial cluster centers.
(b) Assignment: for each observation $x_{i}$ and each cluster center $\mu_{j}$, calculate the dynamic time warping (DTW) distance between them (note: DTW distance can be calculated by using a dynamic programming matrix. It takes into account the shape differences between time series and allows for stretching and compression along the time axis [18]. Thus, using DTW can better characterize the distance between time series) as
  (6) $$\begin{array}{c} d_{\min}=\min(DTW\left(x_{i},\mu_{j-1}\right),DTW\left(x_{i-1},\mu_{j}\right),\\ DTW\left(x_{i-1},\mu_{j-1}\right)+d\left(x_{i},\mu_{j}\right)). \end{array}$$
(c) Update: for each cluster, calculate the average shape of all observations within that cluster and set it as the new cluster center:
  (7) $$\mu_{j}=\mathrm{mean}\left(x_{i}\right)\;\mathrm{for}\;x_{i}\;\mathrm{in}\;\mathrm{Cluster}\;j.$$
(d) Repeat steps (b) and (c) until the termination condition is met.
(e) Evaluation: evaluate the quality of the clustering using the silhouette coefficient. After completing the clustering, calculate the silhouette coefficient for each vector in the clusters. Taking point *i* as an example, to obtain the silhouette coefficient, we should first calculate the average distance a(i) from the vector to all other points within the cluster it belongs to and the average distance, denoted by b(i), from the vector to all points within different clusters that do not include it. Then, the silhouette coefficient of point *i* is given as:
  (8) $$S\left(i\right)=\frac{b(i)-a(i)}{\max(a(i),b(i))}.$$

Overall, using DTW as the distance calculation method in the K-Means clustering algorithm is more suitable for clustering time series data as it considers the shape variations between time series.

There is a certain degree of similarity between some individual battery cells since high consistency can extend the battery’s service life and ensure the safety of the battery pack’s operation [19]. Those battery cells or battery points also exhibit similar performance during normal charging and discharging processes. Therefore, the K-Means algorithm can be employed to delineate the abnormal characteristics of batteries. When using the K-Means algorithm to process these battery data, the data produced by battery cells/points with similar performances will be grouped together into the same cluster. When one of the batteries/points experiences an abnormality, its performance will be different from the other batteries/points in the original cluster, and eventually an “outlier” behavior occurs, meaning that this battery/point no longer belongs to the previous cluster. In this experiment, K-Means algorithm is used to process battery temperature data. When temperature data are abnormal, thermal runaway is a highly likely result. Therefore, when the clustering results change, a TR warning will be triggered.

**Figure 1:**
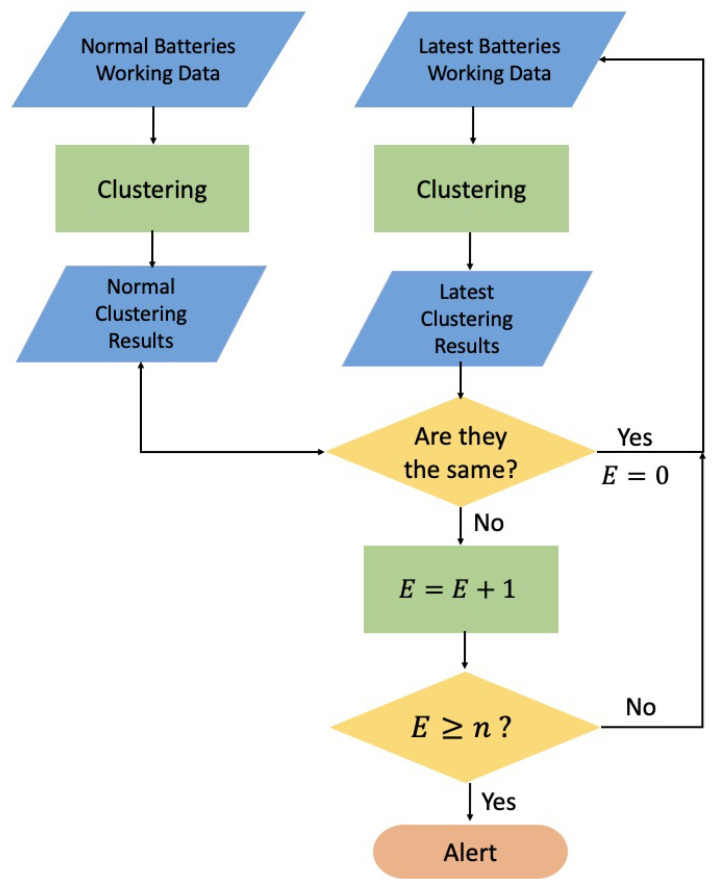
Workflow of battery thermal runaway (TR) warning based on K-Means algorithm.

The algorithm flowchart for TR warning is shown in Figure 1, where *E* represents the number of times a battery is marked as abnormal. Assuming that there is an individual battery cell, the input to the algorithm consists of continuous temperature data from different surfaces of the battery cell. If the subject is a battery pack, the input data are continuous temperature data from different battery cells within the pack. First, the algorithm takes the off-line normal resting, charging, and discharging data of the battery as input. The K-Means clustering algorithm produces three corresponding clustering results, which can be recorded as the “normal clustering results”. Then, real-time working data of the battery cell are use as input, and the clustering algorithm performs clustering on the temperature data from the past *t* seconds, updating the clustering results every second. Each time a clustering result is generated, it is compared to the normal clustering result. If it is consistent with the normal clustering result, the battery is considered normal. If there is a change compared to the normal clustering result, it is recorded as an anomaly. When the battery status is marked as abnormal for *n* consecutive times, it is diagnosed as a prelude to thermal runaway, and the platform issues an alert.

Compared to other data-driven methods, including support vector machines, neural networks, and other derivative methods, the pre-training process of K-Means algorithms is simpler. With less data required, K-Means algorithm can swiftly identify battery features. The reduction in data costs greatly enhances the universality of K-Means algorithms, allowing it to be quickly transferred and utilized in other models.

## 3. A Combined Data-Driven and Model-Driven Algorithm for Battery TR Warning

The preceding section introduced the Bernardi equation and the K-Means clustering algorithm, both of which can individually facilitate early warning of battery thermal runaway. However, in practical applications, both methods have certain limitations. For instance, the determination of the alarm threshold in the Bernardi equation is challenging, leading to a low advance warning; on the other hand, the K-Means algorithm lacks the capability to automatically update the normal clustering results of batteries, resulting in false alarms.

To address the shortcomings of existing studies, this paper proposes a battery thermal runaway warning method that integrates model-driven and data-driven approaches. The algorithm utilizes the K-means algorithm as the data mining component to monitor in real-time the presence of outliers in the battery pack. Upon detecting anomalies, the Bernardi equation is incorporated as the model-driven component to concurrently process battery data and determine whether an alarm is warranted. By leveraging both model and data-driven algorithms simultaneously, this method strikes a balance between an extended alarm lead time and a reduced probability of false alarms.

### 3.1. Algorithm Workflow

**Figure 2:**
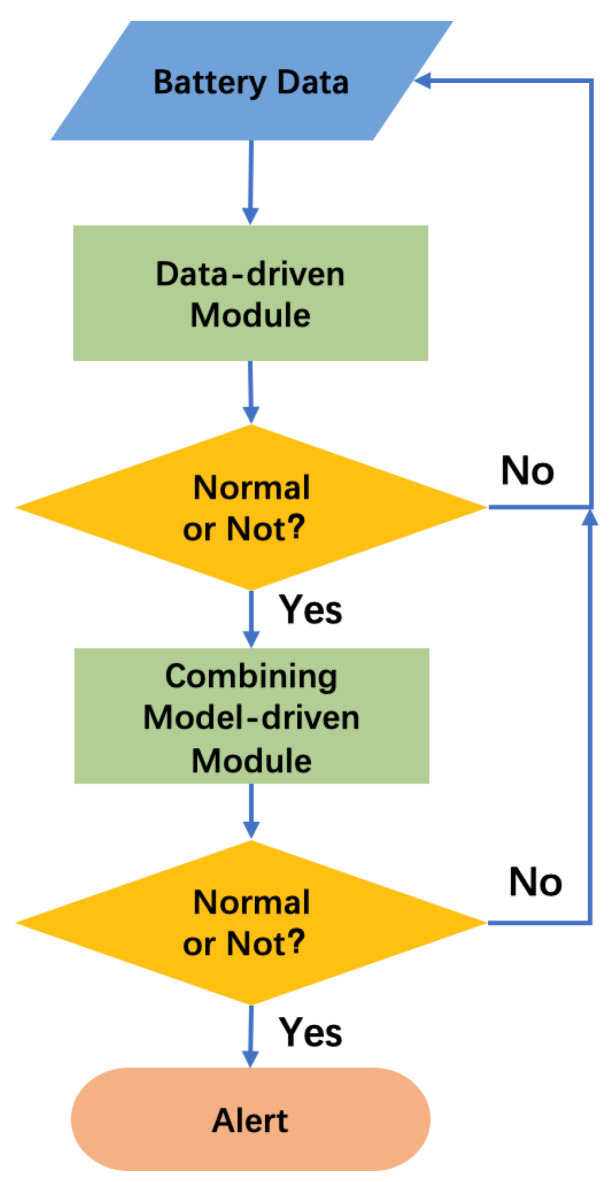
Workflow of the combined data-driven and model-driven algorithm for battery TR warning.

The flowchart of the proposed algorithm is shown in Figure 2. For the most part, only a data-driven module is used to diagnose the working status of batteries, and its specific diagnostic methods have been introduced in Section 2.2. If the data-driven module determines that the battery is normal, it enters the next cycle to receive and process the latest battery data. If the data-driven module reports a warning, the model-driven module will be triggered to assist in predicting the temperature of the battery. At this point, the data-driven module will output the changes in clustering silhouette scores ΔS, while the model-driven module will output the difference between the predicted temperature value and the actual battery cell temperature ΔT. Subsequently, the pre-trained weight results are utilized to aggregate ΔS and ΔT. If the aggregated result surpasses the predetermined threshold, a TR alert is triggered.

The above algorithmic structure can effectively achieve the complementary advantages of data-driven modules and model-driven modules. Specifically, the data-driven module leads the TR alarm, ensuring that the algorithm has a high lead time. At the same time, when anomalies are detected in the data-driven module, the model-driven module and data-driven module analyze battery data in parallel, ensuring the accuracy of the alarm.

However, there are still some parameters yet to be determined in the proposed algorithm, such as the battery entropy coefficient in the Bernardi equation and the weighted ratio of the model-driven module and data-driven module outputs. Therefore, before using the proposed algorithm, we need to conduct pre-training.

### 3.2. Pre-Training

Prior to implementing the proposed double-driven method, we need to confirm the various battery parameters in the Bernardi equation as well as the weighted ratio of the model-driven module and data-driven module outputs.

First, identify the type of battery for monitoring and record the static temperature data of the battery under constant temperature conditions, along with the voltage data of the battery port under pulse current discharge conditions and variable temperature conditions. Then, utilize the obtained data to establish the mapping curve of the battery heat dissipation coefficient, Open Circuit Voltage (OCV) and SOC, as well as the mapping curve of entropy heat coefficient and SOC, to derive the Bernardi equation. The specific method for this establishment will be detailed in Section 4.

**Figure 3:**
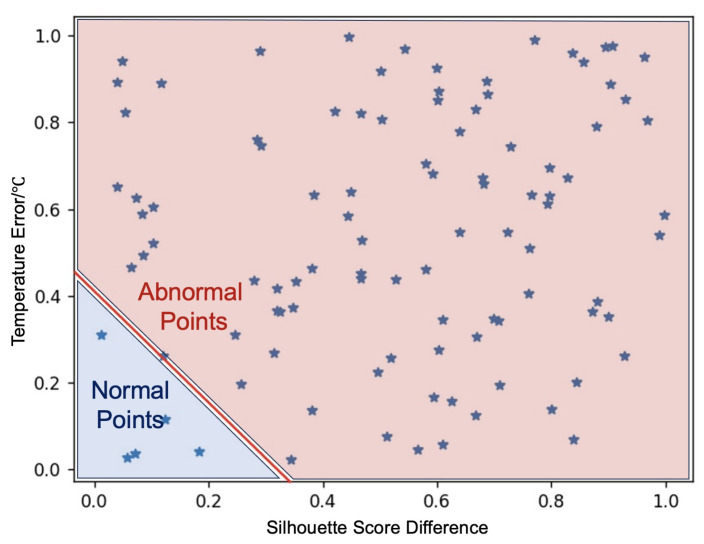
Using SVM to determine the TR alarm threshold.

Subsequently, develop a pre-training model and a data-driven thermal runaway warning algorithm for battery packs using voltage, current, and temperature data from battery packs of the same model under normal operating conditions and thermal runaway conditions. Then, execute the following steps to determine the key parameters for the proposed algorithm.

(A) First, we need to record the different output results of the K-Means algorithm when processing battery pack data under normal and thermal runaway conditions. To achieve this, we employ the K-means algorithm for the time series to process the temperature data of battery pack A, which comprises the same type of battery cells. By utilizing the temperature variations of various battery cells within the battery pack under normal working conditions as input, we obtain the normal clustering results and the corresponding silhouette coefficient for the battery pack’s temperature. In the absence of any abnormalities in the battery cell, the clustering results of the battery pack generally remain unchanged over time.
(B) Apply the K-means algorithm to process the voltage, current, and temperature data of battery pack A under thermal runaway conditions, and output clustering results and silhouette coefficient variation under abnormal conditions.
(C) We also need to record the performance of the Bernardi equation when it deals with the same thermal runaway data. Utilize the Bernardi equation to process the voltage, current, and SOC data of battery pack A under normal operating conditions and thermal runaway conditions, as well as the output temperature data predicted under different conditions. By using the Bernardi equation to process the normal and abnormal data of the same battery pack as in Step B, predict the current temperature value using past voltage and current data and calculate the difference between the output and the actual battery cell temperature.
(D) In this step, we obtain the weighted ratio of the dual-drive algorithm. Through steps A to C, the same data, i.e., battery pack A under normal and thermal runaway conditions, are processed by both the K-Means algorithm and the Bernardi equation. Therefore, we obtain the variation in Silhouette Score output by the K-Means algorithm and the difference between the predicted temperature output by the Bernardi equation and the actual temperature at each moment. As shown in Figure 3, plot each time point into a Cartesian coordinate system, using the output of the data-driven module as the horizontal axis and the output of the model-driven module as the vertical axis. Then, we label each time point to indicate whether it belongs to a normal or abnormal data sequence. Then, we use Support Vector Machines (SVM) [20] to find a straight line that can segment normal and abnormal data. This line represents the alarm threshold of the dual-drive algorithm, which can be expressed as:
  (9) kΔS+bΔT>1,
  where *k* and *b* are constant parameters determined by SVM, ΔS represents the clustering score change value output by data-driven methods, and ΔT denotes the predicted temperature difference output by model-driven methods. When the inequality kΔS+bΔT>1 is satisfied, it indicates a potential risk of thermal runaway in the battery, leading to the issuance of an alarm.

## 4. Experiments

In this section, real battery thermal runaway data are utilized to test the comprehensive performance of the proposed double-driven TR warning algorithm. A model-driven algorithm and data-driven algorithm are also tested to compare their performance differences with the double-driven approach.

### 4.1. Data Source

The data used in this paper are obtained during thermal runaway assessments conducted by a battery manufacturing company, including battery temperature, voltage, and current. The experimental objects encompass two lithium–ion battery cells and two lithium–ion battery packs.

**Figure 4:**
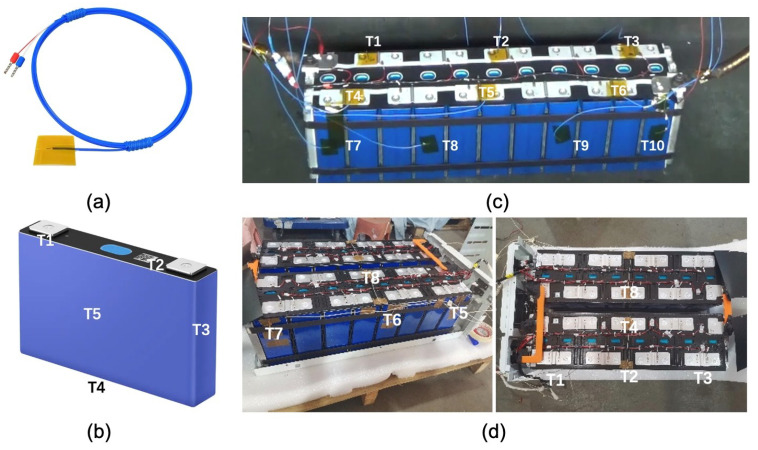
(**a**) Multiple high-temperature resistant thermocouples are used. (**b**) There are 5 measurement points set on the battery cells. (**c**) There are 10 measurement points set on battery group No. 1. (**d**) There are 8 measurement points set on battery group No. 2.

In the experiment, multiple high-temperature resistant thermocouples as shown in Figure 4a are attached to the surface of the battery with insulating tape to measure the temperature. In the case of battery cells, temperature sensors are strategically positioned at the anode, cathode, and three surfaces, as shown in Figure 4b. For battery packs, temperature sensors are placed on the exteriors of 8 to 10 battery cells within each pack, as shown in Figure 4c,d.

In the thermal runaway assessments, the initiation of thermal runaway is facilitated through overcharging and controlled external heating. The experimental procedure unfolds as follows, wherein Step 4 is exclusively executed for the battery cell configuration.

1. The battery cells/battery packs are subjected to an initial charging process.
2. A flat or rod-shaped heating device, with its surface covered in ceramic, is used. The heating device is assembled with the battery cells, directly in contact with them. The size of the heating device does not exceed the heated surface of the test object. Temperature sensors are installed, and the temperature monitoring points are placed on the side farthest from heat conduction, i.e., on the opposite side of the heating device. The sampling interval for temperature data is 0.5 s, with an accuracy ±2 °C. The diameter of the temperature sensor’s tip is less than 1 mm.
3. After fully charging the battery cells using the standard charging method, the cells are continuously charged with a current of 1 Coulomb.
4. After overcharging, the heating device is immediately activated, applying its maximum power to continuously heat the test object. The triggering is stopped when thermal runaway occurs or when the temperature at the monitoring point (opposite the heating surface) reaches 300 °C. The heating device is then turned off.

The criteria for determining thermal runaway is as follows:

(A) The test object experiences a voltage drop.
(B) The temperature at the monitoring point reaches the battery’s protection operating temperature, which is 60 °C.
(C) The temperature rising rate at the monitoring point, dT/dt, is greater than or equal to 1 °C/s.

When both (A) and (C) or (B) and (C) occur, it is considered that a thermal runaway has occurred. The test is terminated when a fire or explosion occurs during the heating process or within one hour after heating.

### 4.2. Pre-Training

Before starting the algorithm testing, we use some battery data to complete the fitting of various parameters in the model-driving module, namely the Bernardi equation.

Firstly, we need to determine the heat dissipation coefficient. Place the fully charged battery in a windless environment to naturally cool down and record the temperature change curve of the battery. According to the Bernardi equation, when the current is 0, the temperature change of the battery satisfies the following equation:

(10) $$Cm\frac{dT}{dt}=h\left(T_{amb}-T\right).$$

By setting $\frac{h}{Cm}$ to $h^{\prime }$, we can obtain:

(11) $$T=T_{amb}+Be^{-h^{\prime }t}$$

**Figure 5:**
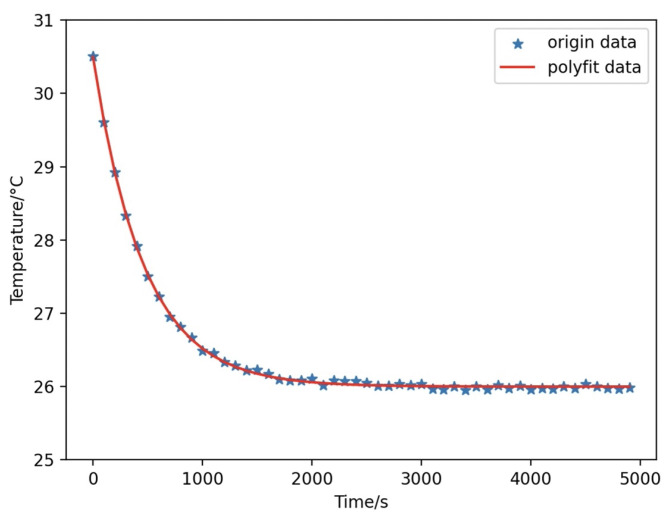
Battery cooling data and the fitted curve.

By substituting the collected battery cooling curve, the least squares method can be used to fit the values of constant *B* and heat dissipation coefficient *h*′. The final result shows that the value of the heat dissipation coefficient *h*′ is about 0.009, and Figure 5 shows the battery cooling data and the fitted curve.

**Figure 6:**
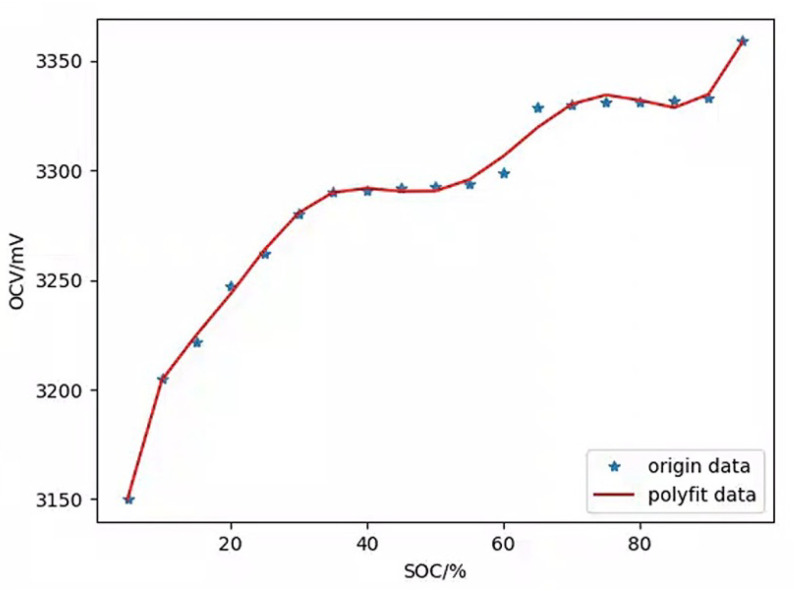
The corresponding curve of SOC and OCV.

Secondly, measure the OCV of a fully charged battery of the same model. Subsequently, discharge 10% of the battery capacity in the form of pulse discharge and measure its OCV again. Repeat the above experiment until the battery is fully discharged. Based on the above data, we can obtain the corresponding curve of SOC and OCV, as shown in Figure 6.

**Figure 7:**
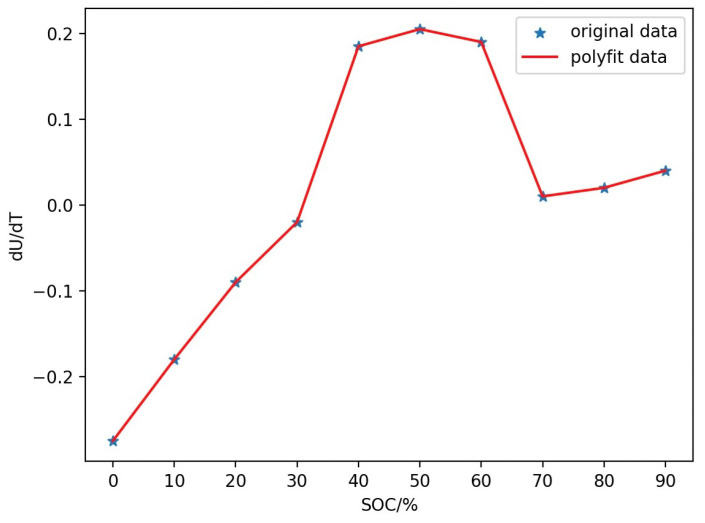
The corresponding curve of entropy heat coefficient and SOC.

Next is the measurement of entropy heat coefficient. Place the battery in a temperature cycling chamber, adjust the temperature to 40 °C, and let it stand for 25 h to ensure a stable open circuit voltage. Finally, the temperature is changed every 3 h in the order of 10, 20, 30, and 40 °C, and the open circuit voltage is measured as a function of temperature and time. After that, the battery is discharged at 10% of its capacity, and it is left at 40 °C for 25 h before continuing to measure the changes in the open-circuit voltage at different temperatures. From this, the open circuit voltage over time curves of the battery at 0–100% SOC can be obtained. Based on the above data, the corresponding curve between entropy heat coefficient and SOC can be obtained, as shown in Figure 7.

**Figure 8:**
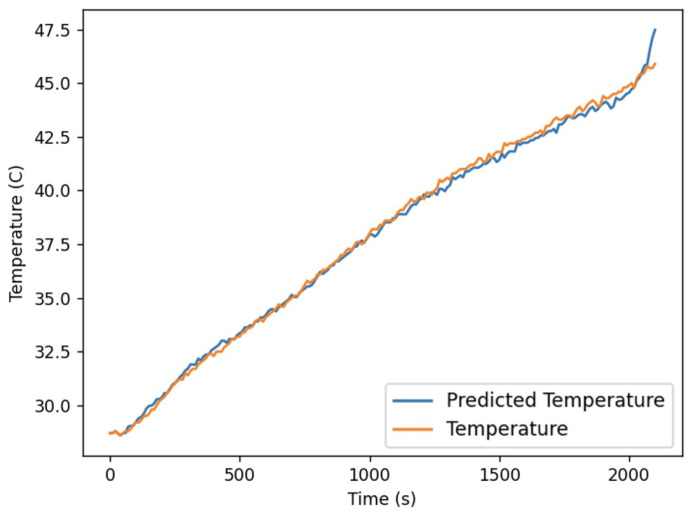
The real and predicting temperature of a charging battery.

At this point, all parameters in the Bernardi equation have been determined and can be used to predict temperature changes during normal charging and discharging of the battery. Taking Figure 8 as an example, the battery is charged at a rate of 1 P. The Bernardi equation accurately estimates the temperature change of the battery, with a maximum error of no more than 0.5 °C. Considering that the temperature sensor may have an error of about 2 °C, the alarm threshold of the model-driven method is set to 3 °C. When the temperature of all measured points exceeds the alarm threshold, the model-driven method will issue an alarm.

Moreover, the alarm thresholds in the dual-drive algorithm can be confirmed, that is, the values of *k* and *b* in Equation (9) are determined to be 2.38 and 3.13, respectively.

### 4.3. Battery Thermal Runaway Prediction

**(1) Results for battery cell cases**

For the thermal runaway detection of battery cell, we collect temperature variation data from five different positions on the surface of a battery cell. In this experiment, two battery cell are tested.

Taking battery cell A as an example, the data-driven method, K-Means algorithm is first used for thermal runaway warning testing. The K-Means algorithm continuously processes the most recent 60 s of battery data and obtains the clustering results under normal operating conditions, as shown in Table 1 (line 1), which remain unchanged before battery overcharging begins. Until 15:19:43, 70 s after the battery begins to overcharge, the battery clustering situation changes as shown in Table 1 (line 2), triggering the battery thermal runaway warning, which occurs approximately 27 min before the actual battery thermal runaway at 15:46:23.

The model-driven method, using the Bernardi equation, is also tested to predict the temperature changes of each battery cell under normal conditions. The alarm remains untriggered until 15:30:48, when the predicted temperature value differs from the actual temperature by more than 3 °C. At this point, there are only 16 min left until the actual occurrence of thermal runaway.

**Figure 9:**
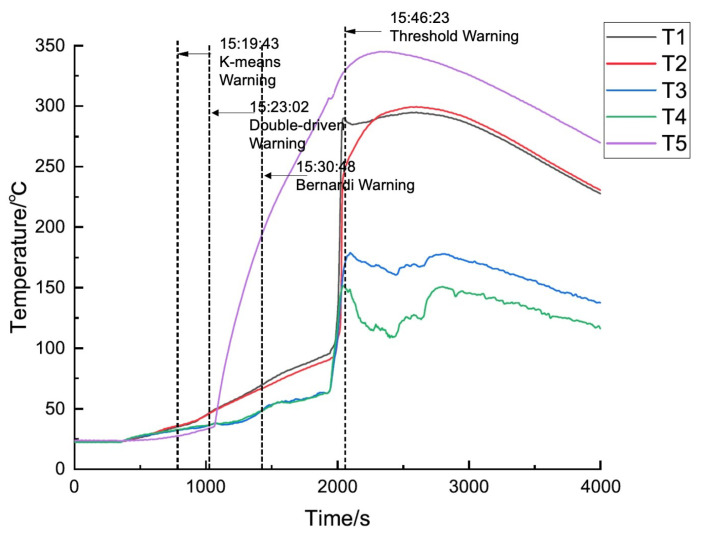
Temperature variation and thermal runaway prediction of battery cell No. 1.

**Figure 10:**
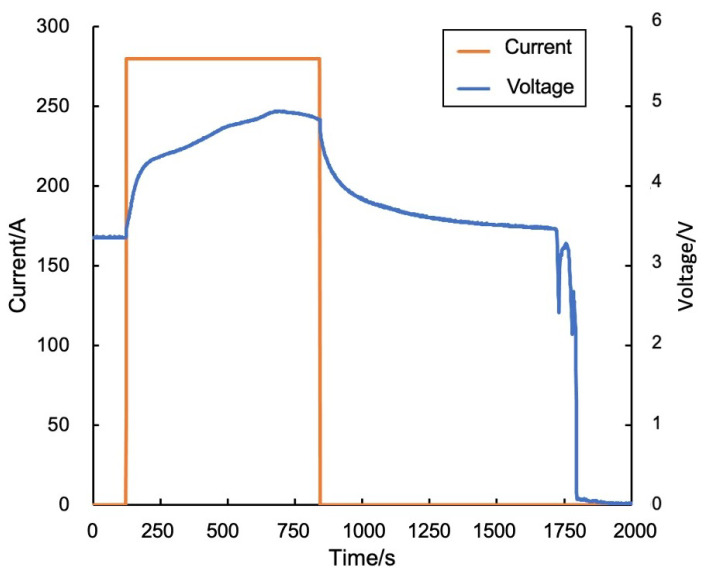
Charging voltage and current of battery cell No. 1.

Finally, there is testing of the proposed algorithm. The proposed method issues a thermal runaway warning at 15:23:02, which is about 23 min earlier than the actual occurrence of thermal runaway. In terms of alarm timing, the performance of the dual-drive method ranks between the data-driven method and the model-driven method. The specific temperature variations and warning results are shown in Figure 9. for the thermal runaway induction period, the changes in charging voltage and current of battery cell No. 1 are shown in Figure 10.

During our experiment with battery B, the data-driven algorithm triggered one false alarm. The normal clustering results are shown in Table 1 (line 3). However, at 9:01:02, the K-Means algorithm triggered a false alarm as the overcharging had not started yet. This is because even under normal usage batteries may undergo internal structural changes due to slow aging, which may lead to changes in clustering results. Until 9:03:28, 50 s after the battery began to overcharge, the battery clustering situation changed again as shown in Table 1 (line 4), triggering another battery thermal runaway warning, which occurred approximately 28 min before the actual battery thermal runaway at 9:32:37.

**Figure 11:**
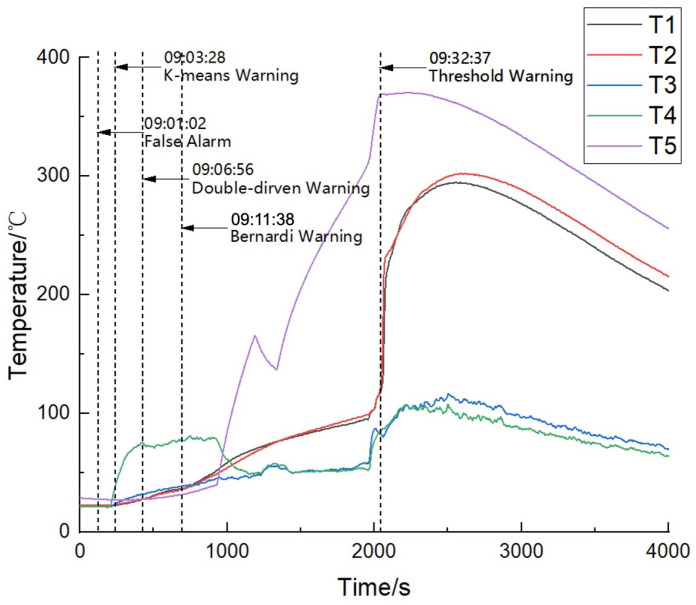
Temperature variation and thermal runaway prediction of battery cell No. 2.

**Figure 12:**
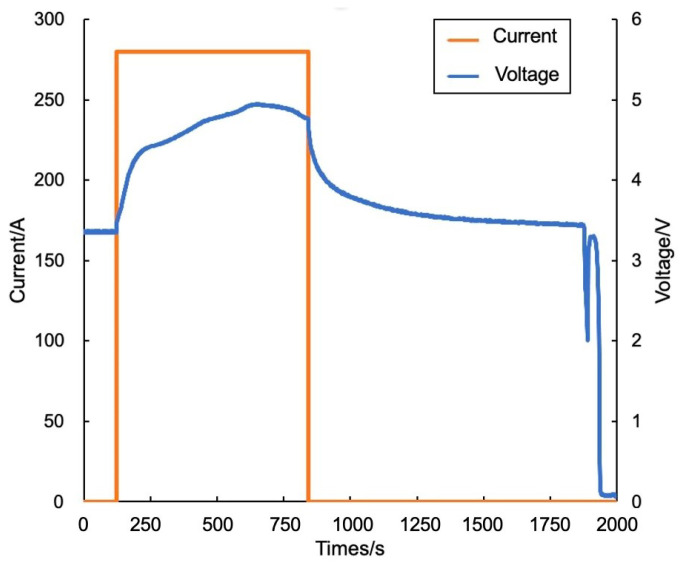
Charging voltage and current of battery cell No. 2.

In addition, the model-driven approach and the proposed algorithm both issued thermal runaway warnings 21 min and 26 min in advance, respectively, and neither triggered false alarms. In terms of alarm accuracy, the dual-drive algorithm successfully avoided the false alarm that occurs when applying the data-driven method. The specific temperature variations and warning results are shown in Figure 11. For the thermal runaway induction period, the changes in charging voltage and current of battery cell No. 2 are shown in Figure 12.

**(2) Results for battery group cases**

The method proposed in this paper is also applicable for thermal runaway prediction in battery packs. Collect temperature data from individual battery cells within the battery packs as input for the algorithm. Specifically, battery pack A comprises 10 individual battery cells, while battery pack B contains 8 individual battery cells.

Taking battery group A as an example, the data-driven method, K-Means algorithm can first be used for thermal runaway warning testing. The K-Means algorithm continuously processes the most recent 60 s of battery data and obtains the clustering results under normal operating conditions, as shown in Table 2 (line 3). However, in our experiment, at 11:11:15 the K-Means algorithm triggered a false alarm as the overcharging had not started yet. Until 11:13:59, 97 s after the battery began to overcharge, the battery clustering situation changed again as shown in Table 2 (line 4), triggering another battery thermal runaway warning, which occurred approximately 29 min before the actual battery thermal runaway at 11:43:16.

The model-driven method, Bernardi equation can also be tested to predict the temperature changes of each battery cell under normal conditions. In our experiment, the alarm remained untriggered until 11:24:10, when the predicted temperature value differed from the actual temperature by more than 3 °C. At this point, there were about 19 min left until the actual occurrence of thermal runaway.

**Figure 13:**
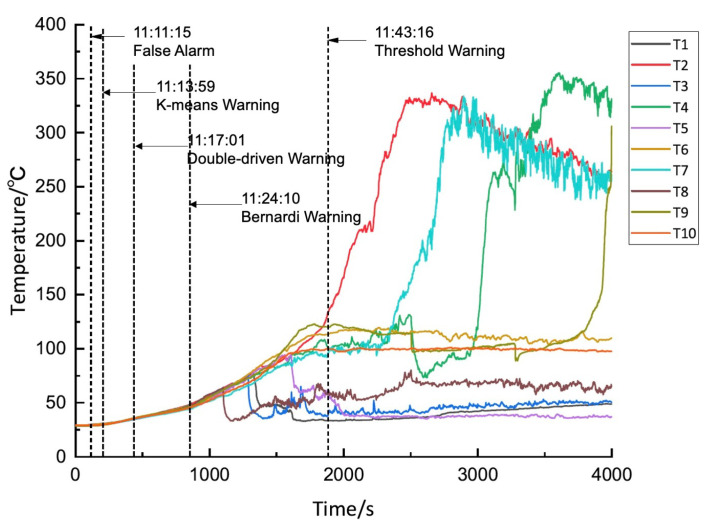
Temperature variation and thermal runaway prediction of battery group No. 1.

**Figure 14:**
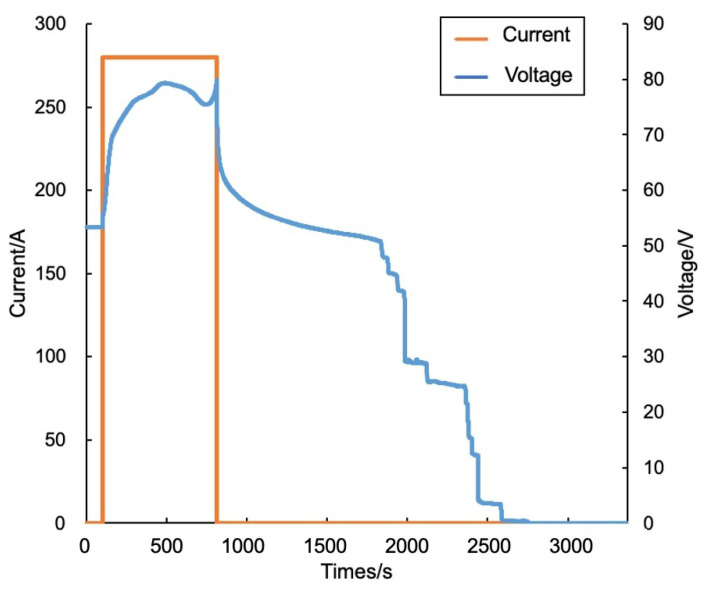
Charging voltage and current of battery group No. 1.

Finally, there is testing of the dual-drive algorithm. In our experiment, the dual-drive method issued a thermal runaway warning at 11:17:01, which was about 26 min earlier than the actual occurrence of thermal runaway. In terms of alarm timing, the performance of the dual-drive method ranks between the data-driven method and the model-driven method. In terms of alarm accuracy, the dual-drive algorithm successfully avoided the false alarm that occurs when applying the data-driven method. The specific temperature variations and warning results are shown in Figure 13. For the thermal runaway induction period, the changes in charging voltage and current of battery group No. 1 are shown in Figure 14.

**Figure 15:**
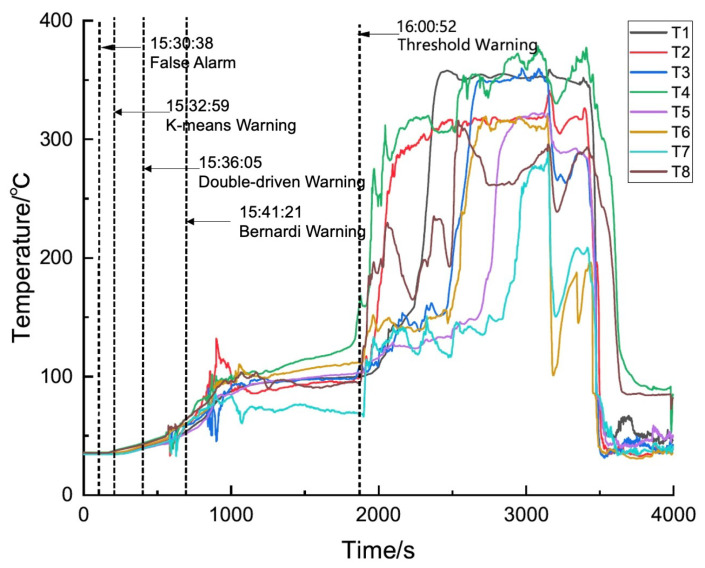
Temperature variation and thermal runaway prediction of battery group No. 2.

**Figure 16:**
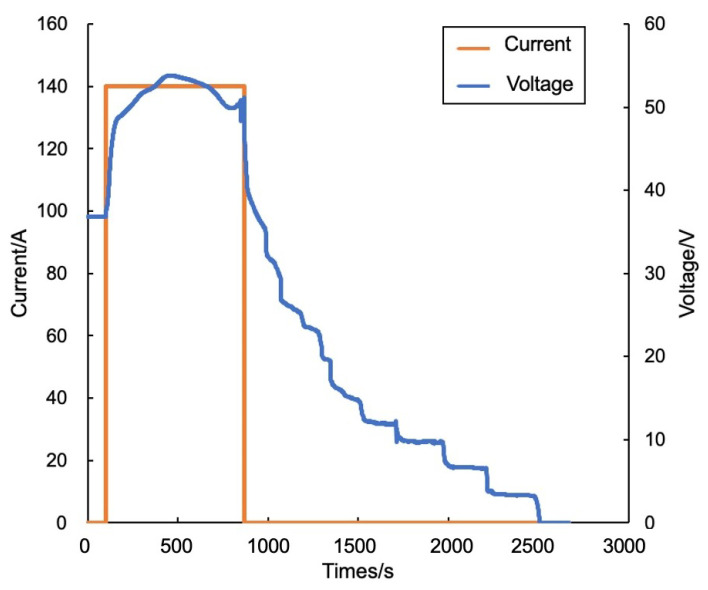
Charging voltage and current of battery group No. 2.

In the experiment of battery pack B, the data-driven method also triggered one false alarm, ultimately achieving a thermal runaway warning 28 min in advance. The clustering output results varied as shown in Table 2 (line 3 and line 4). In addition, the model-driven approach and the proposed algorithm issued thermal runaway warnings 19 min and 24 min in advance, respectively, without triggering any false alarms.The specific temperature variations and warning results are shown in Figure 15. For the thermal runaway induction period, the changes in charging voltage and current of battery group No. 2 are shown in Figure 16.

From the above four sets of experiments, it can be observed that the proposed double-driven TR warning algorithm performs excellently in both individual battery thermal runaway and group battery thermal runaway warnings. It can issue thermal runaway warnings approximately 25 min before thermal runaway occurs, which is more timely than model-driven method. Meanwhile, compared to data-driven methods, the dual-drive algorithm greatly improves the accuracy of early warning, reducing the false alarm rate by 100% in this experiment.

Finally, we compared several performance indicators of the proposed method with other TR early warning methods in recent years, as shown in Table 3. It can be found that the dual-drive algorithm performs well in terms of warning lead time, warning accuracy, applicable TR types, applicable battery types, and training difficulty, fully demonstrating the advantages of the proposed algorithm.

## 5. Conclusions

The purpose of this study was to develop a battery risk diagnosis method based on both model and data-driven approaches, aiming to improve the recognition rate of battery abnormal states and the accuracy of thermal runaway warning. The core of the paper lies in combining the advantages of data-driven and model-driven methods, and achieving early diagnosis of battery thermal runaway behavior by real-time monitoring of outlier cells and battery data in the battery pack.

In the proposed algorithm, a K-Means clustering algorithm continuously outputs the Silhouette Coefficient variation of the battery temperature data, while the Bernardi equation continuously outputs the difference between the predicted temperature and the actual temperature. Through SVM, suitable weighted ratios for the outputs of the two modules are found, resulting in accurate thermal runaway alarm thresholds. The effectiveness of the proposed method was verified through four sets of battery thermal runaway experimental data. This method can issue a warning about 25 min before thermal runaway occurs, and achieved 100% accuracy during the experimental process.

In summary, the proposed algorithm has successfully achieved early diagnosis and early warning of battery thermal runaway behavior. Compared to traditional single data-driven or model-driven methods, the dual-drive algorithm in this study significantly improved the accuracy of early warning and reduced the probability of false alarms, providing a new technological means for the field of battery safety management.

## Figures and Tables

**Table 1:** Clustering result of battery cells.

Cluster Number	Normal Result No. 1	Abnormal Result No. 1	Normal Result No. 2	Abnormal Result No. 2
1	T1, T2	T1	T1, T2	T1, T2
2	T3, T4	T2, T3	T3, T4	T3
3	T5	T4	T5	T4
4	/	T5	/	T5

**Table 2:** Clustering result of battery groups.

Cluster Number	Normal Result No. 1	Abnormal Result No. 1	Normal Result No. 2	Abnormal Result No. 2
1	T1, T2, T3, T4, T5, T6	T1	T1	T1, T3, T5, T7
2	T7, T10	T2, T3, T4, T5, T6	T2, T4, T6, T8	T2, T6
3	T8, T9	T7, T8, T9, T10	T3, T5, T7	T4, T8

**Table 3:** The proposed method compared with other TR warning algorithm.

Method	Warning Lead Time	Warning Accuracy	Applicable TR Types	Applicable Battery Types	Training Difficulty
[7]	50–300 s after the error happens	higher that 90%	ISC, overheat	battery cell	easy
[8]	100 s after the error happens	no discussion	impedance fault	battery cell	easy
[10]	28 min before TR	error rate 0.28%	all types	battery cell	very complex
[12]	90 min before TR	no discussion	all types	battery group	easy
the proposed method	60 s after the error happens and 25 min before TR	100% in experiments	all types	battery cell and battery group	easy

## Data Availability

Data used in this paper is unavailable due to privacy restrictions.
